# Association of main meal frequency and skipping with metabolic syndrome in Korean adults: a cross-sectional study

**DOI:** 10.1186/s12937-023-00852-x

**Published:** 2023-05-11

**Authors:** Haeun Park, Dayeon Shin, Kyung Won Lee

**Affiliations:** 1 Department of Food and Nutrition, Inha University, 100 Inha-ro, Michuhol-gu, Incheon, 22212 Republic of Korea; 2 Department of Home Economics Education, Korea National University of Education, 250 Taeseongtabyeon-ro, Heungdeok-gu, Cheongju, 28173 Republic of Korea

**Keywords:** Meal frequency, Meal skipping, Metabolic syndrome, Korean adults, KNHANES

## Abstract

**Background:**

Reduced meal frequency can increase the risk of metabolic syndrome (MetS). However, limited studies have examined the association between meal frequency and skipping meals with MetS. This study aims to analyze the association between main meal frequency and meal skipping with MetS in Korean adults aged ≥ 19 years.

**Methods:**

In this study, we included data from 22,699 Korean adult participants from the 2016–2020 Korea National Health and Nutrition Examination Survey (KNHANES). The 24-h dietary recall method was used to classify the participants into three groups based on main meal frequency (one, two, or three meals per day) and seven groups based on the type of main meal they skipped. Multivariable logistic regression analysis was conducted to determine the association between main meal frequency and the types of main meals skipped with the odds of MetS and its associated components. Appropriate estimates were accounted for using sampling weights, stratification, and clustering.

**Results:**

The prevalence of MetS in the study population was 33.8%. The average age of the participants was 47.2 years with 42.6% being men, and 57.4% being women. Men who consumed two meals per day had higher odds of MetS than those who consumed three meals per day (odds ratio [OR] 1.16, 95% confidence interval [CI] 1.01–1.33). Women who consumed two meals per day, and skipped breakfast had increased odds of having elevated fasting blood glucose levels (OR 1.18, 95% CI 1.02–1.35), and elevated triglycerides (OR 1.19, 95% CI 1.02–1.39). However, women who skipped dinner had reduced odds of having elevated fasting blood glucose levels (OR 0.74, 95% CI 0.58–0.94).

**Conclusions:**

Our findings suggest that meal frequency and the type of main meal skipped may be associated with MetS and emphasize the importance of consuming breakfast to prevent MetS.

## Background

Metabolic syndrome (MetS) is a complex disorder characterized by a cluster of conditions that increase the risk for cardiovascular disease and type 2 diabetes, partly because of a common underlying pathophysiology [1]. Previous research has shown that various risk factors associated with cardiovascular disease often coexist in individuals with MetS and substantially increase the incidence of cardiovascular disease [1]. In 1988, the World Health Organization (WHO) [2] first defined MetS as a cluster of metabolic abnormalities including insulin resistance, obesity, dyslipidemia, and hypertension [3, 4]. The criteria proposed by the National Cholesterol Education Program Adult Treatment Panel (NCEP-ATP III) in 2001 represent the most widely used definitions of MetS and include hyperglycemia, insulin resistance, visceral obesity, atherogenic dyslipidemia, and hypertension [4]. The International Diabetes Foundation published the new criteria in 2005 [5], which further clarified that the distribution of weight and waist circumference norms varies by population, ethnicity, and nationality in individuals with abdominal obesity [4].

South Korea has experienced rapid socioeconomic growth in recent decades, resulting in lifestyle changes that have considerably increased the prevalence of MetS risk factors, including obesity and type 2 diabetes [6]. The prevalence of MetS is increasing in Korea, particularly among men [7]. According to the Korean Society of Cardiometabolic Syndrome, the prevalence of MetS among Korean adults was approximately 23% in 2018 and varied according to sex (27.9% in men and 17.9% in women). Furthermore, a high prevalence of MetS (45.3%) has been reported among older adults aged above 65 years [8]. According to the WHO, risk factors of MetS are associated with an increased risk of chronic diseases [8, 9]. To effectively address this issue and ultimately prevent a global epidemic, the NCEP-ATP III recommends dietary and lifestyle changes [3].

Diet plays a crucial role in the prevention and management of chronic diseases, such as MetS, obesity, and diabetes [10, 11]. Recent evidence indicates that Korean dietary habits have become characterized by irregular eating patterns such as meal skipping and reduced meal consumption frequency [12]. These dietary habits have been linked to several chronic diseases. A study that assessed the dietary habits of 19-year-old Korean adults revealed that men who consumed two or fewer meals per day had significantly higher odds of MetS than those who consumed three meals per day (odds ratio [OR] = 1.37, 95% confidence interval [CI] = 1.12–1.67) [12]. Similarly, a study conducted among US adults aged 40–50 years found that eating only one meal per day without calorie restriction for two months was associated with elevated fasting glucose levels and impaired glucose tolerance [13]. A European study assessing participants aged 45–75 years found that those who consumed six or more meals per day had lower total and low-density lipoprotein cholesterol levels than those who consumed two meals per day [14]. Based on the findings from the Northern Finland Birth Cohort (NFBC) in 1986, Jääskeläinen et al. [15] found that reduced meal frequency negatively affects appetite control, resulting in increased hunger and food intake, which in turn leads to the development of chronic diseases. Conversely, adherence to regular eating patterns, such as eating three meals per day has beneficial effects on metabolic profiles. In a study assessing individuals aged above 60 years, consumption of three meals per day was associated with a lower prevalence of MetS (OR = 0.63, 95% CI = 0.45–0.88) and insulin resistance (OR = 0.70, 95% CI = 0.50–0.97) than those among individuals consuming one or two meals per day [16]. These findings reveal that increased meal frequency can decrease hunger and maintain optimal insulin concentrations during intermeal periods by preventing large drops in plasma glucose levels between meals [17].

Skipping meals is associated with several adverse health conditions [18–20]. In a cross-sectional study evaluating 3,864 adults aged 20–64 years, skipping breakfast was associated with an increased odds of MetS [21]. In children, skipping meals is associated with an increased risk of obesity [22]. In a study examining the findings of the NFBC of 1986 and Greek adolescents, an inverse relationship was identified between eating breakfast and the odds of overweight (OR = 0.72, 95% CI = 0.54–0.95) and obesity (OR = 0.73, 95% CI = 0.53–0.97) [23]. Skipping breakfast increases the rate of obesity and fasting blood insulin and cholesterol levels among both adults and children, resulting in abnormal blood metabolism [24, 25]. In a study examining the dietary habits of Finnish adolescents, participants with consumption of five meals per day showed a reduced odds of becoming overweight (OR = 0.47, 95% CI = 0.34–0.65) and obese (OR = 0.57, 95% CI = 0.41–0.79) in both men and women compared to those with consumption of four meals per day, skipping breakfast [15]. Although there is limited data suggesting that skipping lunch and dinner can cause metabolic abnormalities in Korean adults, some studies have noted fewer abnormalities in those who skipped dinner compared to those who skipped breakfast [26–29].

Several studies have reported that the frequency of eating and skipping meals is associated with the risk of metabolic abnormalities. However, no previous studies have established whether the frequency of eating main meals and skipping patterns of main meals affect the prevalence of MetS in Korean adults. Therefore, in the present study, we aim to investigate the association between main meal frequency and meal skipping patterns with MetS and its associated components in Korean adults aged ≥ 19 years using data from the Korea National Health and Nutrition Examination Survey (KNHANES).

## Methods

### Data source and study participants

Data from the KNHANES were used to investigate the association between the frequency of consuming main meals and types of main meals skipped with MetS and its associated components among Korean adults aged ≥ 19 years. The KNHANES is a nationwide survey conducted to examine the health status, health-related behaviors, prevalence of chronic diseases, and dietary intake in Koreans. This study used recently released data collected through health interviews, examinations, and nutritional surveys. The KNHANES is an annual cross-sectional study, with 24,269 people participating in the 7th KNHANES (2016–2018) and 15,469 participating in the 8th KNHANES (2019–2020). The samples are representative of the total non-institutionalized population living in Korea, excluding those residing in nursing homes or military bases.

**Fig. 1:**
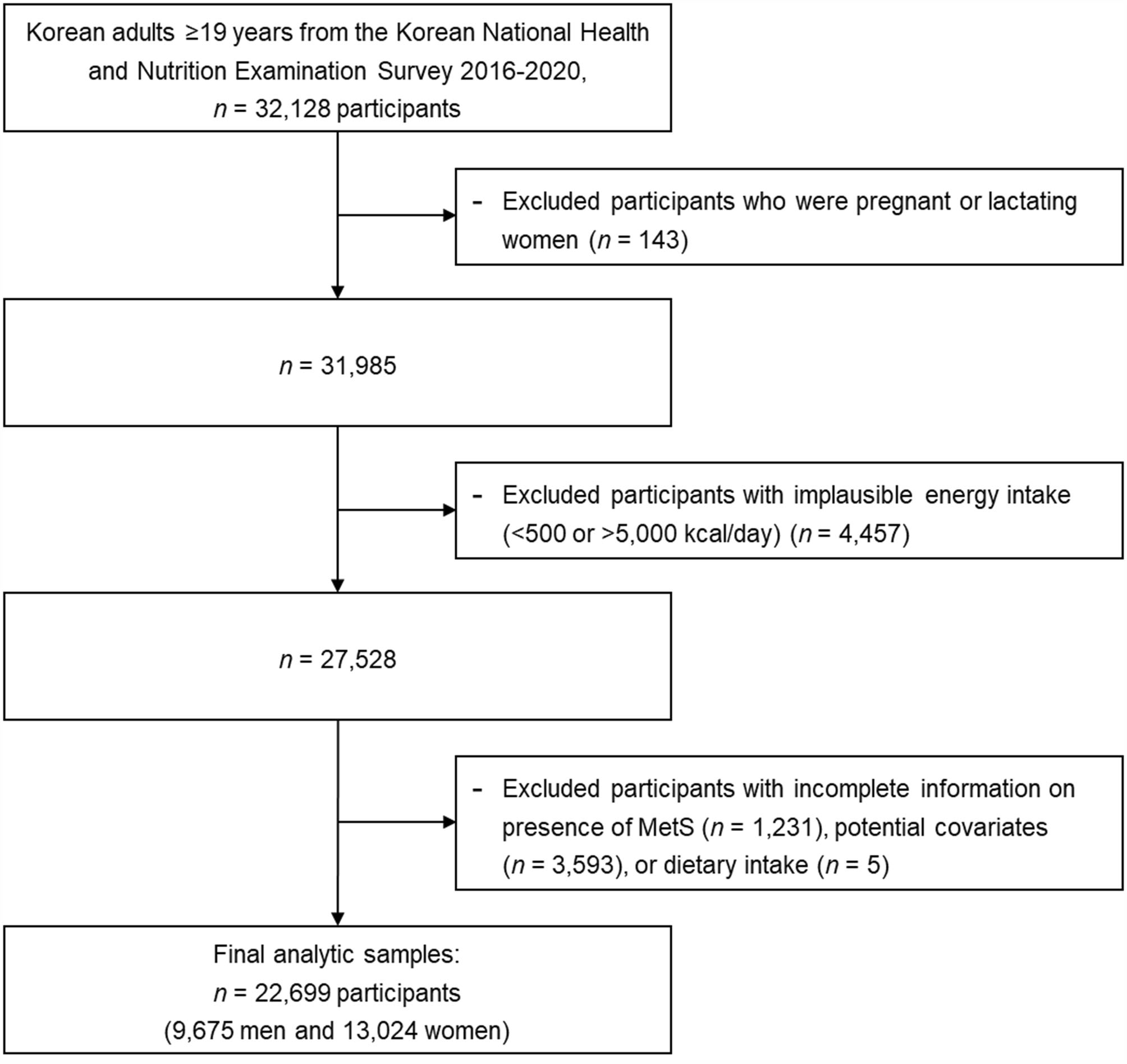
Flow diagram of the study population

A total of 32,128 participants aged ≥ 19 years were included in the KNHANES between 2016 and 2020. The following exclusion criteria were applied: pregnancy or lactation (n = 143), implausible energy intake (< 500 kcal/day or > 5,000 kcal/day) (n = 4,457), insufficient information regarding covariates (n = 3,593), presence of MetS (n = 1,231), and dietary information (n = 5) (Fig. 1). Finally, this study included 22,699 Korean adults (9,675 men and 13,024 women).

### Frequency of main meals and patterns of main meals skipped

The nutrition survey of the KNHANES included data on the status of dietary life, dietary supplements, nutritional knowledge, food safety, and food intake one day before the survey (24-h recall method) and was conducted by visiting targeted households. Among the dietary variables collected, the frequency of main meals per day and types of main meals skipped were defined using single 24-h dietary recall data. During the 24-h dietary recall, participants were asked to list all food and beverage items consumed in the previous 24 h and the eating episodes (breakfast, lunch, dinner, and snacks) during which each item was consumed. Eating episodes such as breakfast, lunch, and dinner were considered the main meals. The participants answered whether they consumed breakfast, lunch, or dinner, regardless of the intake time. Some participants ate more than three meals a day, but the additional intake of food over the three main meals was considered snack intake. The frequency of main meals per day was classified into three categories: three main meals per day, two main meals per day, and one main meal per day. To estimate the odds of MetS based on the type of main meal skipped, we further subdivided the frequency of main meals per day into seven categories: three main meals per day (breakfast, lunch, and dinner), two main meals per day (lunch and dinner, breakfast and dinner, or breakfast and lunch), or one main meal per day (breakfast, lunch, or dinner).

### Definition of MetS and its associated components

The KNHANES examination data were used to obtain anthropometric values corresponding to MetS associated components. The examination survey is conducted annually through direct measurements and sample analysis. As this was a cross-sectional study, the first anthropometric measurement was used to define MetS. Participants with three or more risk factors were defined as having MetS based on the criteria of the NCEP-ATP III and the Korean Society for the Study of Obesity. Abdominal obesity was defined as a waist circumference ≥ 90 cm for men and ≥ 85 cm for women. Elevated triglyceride levels were defined as triglyceride levels ≥ 150 mg/dL. Low levels of high-density lipoprotein (HDL) cholesterol were defined as HDL cholesterol levels < 40 mg/dL in men and < 50 mg/dL in women. Elevated blood pressure was defined as systolic blood pressure ≥ 130 mmHg, diastolic blood pressure ≥ 85 mmHg, being under treatment for physician-diagnosed hypertension, or having a previous history of physician-diagnosed hypertension. Elevated fasting blood glucose was defined as a fasting blood glucose level ≥ 100 mg/dL, undergoing treatment for physician-diagnosed type 2 diabetes mellitus, or a previous history of physician-diagnosed type 2 diabetes mellitus.

### Covariate justification

Covariates were adjusted to examine the independent association between the daily main meal frequency and MetS. Meal frequency varies according to age, and there is a significant difference in food intake among individuals with varying levels of education [30, 31]. Previous studies have reported that lower income levels are associated with lower meal frequency and food intake, that intake patterns differ according to marital status, and that mealtime behavior changes according to household type [32–34]. Furthermore, meal frequency has been associated with alcohol and tobacco consumption, physical activity, and skipping breakfast. The frequency of habitual meals is also associated with energy intake and is inversely associated with body mass index (BMI) [35–38]. Therefore, age, education level, household income level, occupation, marital status, household type, region, alcohol consumption, smoking status, regular physical activity, BMI, and total energy intake were used as adjustment variables in this study.

### Statistical analysis

The SAS (Statistical Analysis System) version 9.4 (SAS Institute, Cary, NC, USA) was used for all statistical analyses, and a two-tailed *P*-value < 0.05 was deemed statistically significant. As recommended by the KNHANES analytical guidelines, PROC SURVEY procedures with sample weights, strata, and primary sampling units were applied to account for the complex survey design [39].

To compare general characteristics according to main meal frequency, categorical variables were analyzed using the chi-square test, and continuous variables were analyzed using analysis of variance (ANOVA). Categorical variables were presented as frequencies with weighted percentages, and continuous variables were reported as means ± standard errors. The frequency of the main meal intake was treated as a categorical variable. Multivariable logistic regression analysis was performed to determine the association between the frequency of main meals, the types of main meals skipped, and the odds of MetS and its associated components. The results were presented as ORs and 95% CIs after adjusting for the following potential covariates: age (years, continuous), educational level (elementary school, middle school, high school, or college or higher), household income level (lower middle, middle, upper middle, or highest), occupation (yes or no), marital status (married or single), household type (single-person or multi-person households), region (urban or rural), alcohol consumption (none, moderate, or high), smoking status (never, past, or current), regular physical activity (yes or no), BMI (kg/m^2^, continuous), and total energy intake (kcal, continuous). All analyses were performed separately for men and women. When comparing general characteristics by sex, lifestyle variables, such as smoking status and dining out rate, which can affect health, showed significant differences. In addition, since endocrine factors, including hormones, such as estrogen and testosterone, have sex-dependent effects, the analysis was performed separately according to sex.

## Results

### General characteristics according to the main meal frequency

Table 1 provides a summary of the general characteristics of the study participants according to the frequency of main meal intake. The prevalence of MetS in the study population was 33.8%, with an average participant age of 47.2 years; 42.6% were men, and 57.4% were women. Individuals who consumed three main meals per day tended to be older, have a higher household income, and have a lower BMI than those who consumed one main meal per day. They were also more likely to be employed, married, and live in rural areas but less likely to be highly educated and current smokers.

**Table 1:** General characteristics of study participants according to main meal frequency

	Men (n = 9,675)				Women (n = 13,024)			
	Main meal frequency (meals/day)			*p*-value	Main meal frequency (meals/day)			*p*-value
	3 (n = 6,502)	2 (n = 2,907)	1 (n = 266)		3 (n = 8,681)	2 (n = 3,979)	1 (n = 364)	
Age, years	51.0 ± 0.3^1)^	39.6 ± 0.3	33.7 ± 0.8	< 0.0001^3)^	51.9 ± 0.2	42.2 ± 0.3	35.8 ± 0.8	< 0.0001
Education				< 0.0001				< 0.0001
≤Elementary	923 (9.2)^2)^	152 (3.0)	9 (1.8)		2,156 (18.9)	522 (9.1)	36 (5.2)	
Middle school	794 (8.8)	187 (4.7)	6 (1.4)		1,062 (10.4)	338 (7.0)	30 (7.1)	
High school	1,794 (27.2)	794 (25.2)	75 (28.3)		2,245 (27.5)	1,131 (28.2)	92 (23.1)	
≥College	2,991 (54.8)	1,774 (67.0)	176 (68.5)		3,218 (43.2)	1,988 (55.7)	206 (64.6)	
Household income				0.0001				0.0002
Lowest	1,042 (11.9)	317 (8.9)	41 (11.5)		1,562 (14.1)	521 (10.8)	41 (8.9)	
Lower middle	1,244 (16.7)	465 (14.6)	50 (17.0)		1,672 (17.6)	732 (17.1)	62 (16.2)	
Middle	1,277 (20.3)	618 (21.5)	52 (20.0)		1,719 (20.7)	871 (22.3)	79 (21.7)	
Upper middle	1,362 (23.0)	760 (27.5)	67 (27.4)		1,811 (22.7)	944 (25.3)	93 (28.2)	
Highest	1,577 (28.1)	747 (27.5)	56 (24.1)		1,918 (25.0)	911 (24.5)	89 (24.9)	
Occupation				0.0025				< 0.0001
Yes	1,983 (25.7)	717 (24.1)	92 (34.5)		4,324 (48.6)	1,722 (42.7)	145 (37.9)	
No	4,519 (74.3)	2,190 (75.9)	174 (65.5)		4,357 (51.4)	2,257 (57.3)	219 (62.1)	
Marital status				< 0.0001				< 0.0001
Married	5,671 (81.1)	1,881 (58.1)	103 (32.5)		8,003 (88.7)	3,033 (69.1)	227 (53.9)	
Single	831 (18.9)	1,026 (41.9)	163 (67.5)		678 (11.3)	946 (30.9)	137 (46.1)	
Household type				< 0.0001				0.3923
Single-person households	655 (8.8)	433 (13.2)	54 (17.8)		1,197 (10.0)	495 (9.5)	50 (11.7)	
Multi-person households	5,847 (91.2)	2,474 (86.8)	212 (82.2)		7,484 (90.0)	3,484 (90.5)	314 (88.3)	
Region				< 0.0001				< 0.0001
Urban	5,085 (84.1)	2,453 (89.1)	232 (92.4)		6,977 (85.7)	3,406 (89.9)	304 (88.2)	
Rural	1,417 (15.9)	454 (10.9)	34 (7.6)		1,704 (14.3)	573 (10.1)	60 (11.8)	
Alcohol consumption				0.0130				< 0.0001
None	2,095 (30.6)	815 (26.6)	73 (26.2)		5,543 (60.5)	2,021 (46.9)	141 (34.7)	
Moderate	2,224 (37.0)	1,044 (38.4)	100 (40.7)		2,351 (29.7)	1,340 (37.2)	150 (43.6)	
High	2,183 (32.4)	1,048 (35.0)	93 (33.1)		787 (9.7)	618 (15.9)	73 (21.7)	
Smoking status				< 0.0001				< 0.0001
Never	1,557 (25.8)	741 (28.8)	76 (31.1)		8,010 (91.6)	3,303 (82.0)	269 (76.7)	
Past smokers	3,220 (45.9)	949 (29.9)	61 (21.4)		420 (5.0)	342 (8.9)	36 (10.5)	
Current smokers	1,725 (28.3)	1,217 (41.3)	129 (47.6)		251 (3.4)	334 (9.1)	39 (12.9)	
Body mass index, kg/m^2^	24.5 ± 0.04	24.9 ± 0.1	25.0 ± 0.3	< 0.0001	23.3 ± 0.1	23.2 ± 0.1	22.8 ± 0.2	0.0232
Physical activity				0.2819				0.6499
Yes	3,539 (50.6)	1,509 (48.9)	126 (46.8)		5,150 (56.8)	2,361 (56.1)	264 (54.4)	
No	2,963 (49.4)	1,398 (51.1)	140 (53.2)		3,530 (43.2)	1,618 (43.9)	160 (45.6)	

^1)^ Continuous variables are presented as means ± standard errors

^2)^ Categorical variables are presented as frequencies (weighted %)

^3)^*P*-values based on analysis of variance for continuous variables and chi-square tests for categorical variables

### Nutrient intake and eating behaviors of study participants according to main meal frequency

Table 2 presents the nutrient intake and eating behaviors of the study participants according to the frequency of the main meal intake. Considering both men and women, individuals consuming three main meals per day had a higher intake of energy, carbohydrates, protein, and fiber; a higher proportion of energy from breakfast; a lower proportion of energy from dinner and snacks than those consuming one main meal per day (all *P* < 0.05). In addition, participants who consumed three main meals per day tended to have a longer eating duration, a greater number of meal episodes, lower energy intake from eating out, and higher adherence to healthy diet practices than those who consumed one main meal per day (all *P* < 0.05). As the main meal frequency decreased from three to two, the percentage of energy from snacks and eating out increased significantly in both men and women. Men and women who consumed three main meals were more likely to adhere to a healthy diet compared to those who had one main meal.

**Table 2:** Dietary intake of study participants according to main meal frequency

	Men (n = 9,675)				Women (n = 13,024)			
	Main meal frequency (meals/day)			*p*-value	Main meal frequency (meals/day)			*p*-value
	3 (n = 6,502)	2 (n = 2,907)	1 (n = 266)		3 (n = 8,681)	2 (n = 3,979)	1 (n = 364)	
Energy, kcal/day	2,221.2 ± 12.4^1)^	2,021.8 ± 17.5	1,548.1 ± 51.1	< 0.0001^3)^	1,709.3 ± 8.6	1,511.9 ± 11.1	1,217.5 ± 32.9	< 0.0001
Carbohydrates, g/day	347.2 ± 1.8	293.7 ± 2.5	210.6 ± 6.6	< 0.0001	276.0 ± 1.4	227.9 ± 1.7	180.6 ± 5.0	< 0.0001
Protein, g/day	87.6 ± 0.6	82.7 ± 0.9	65.9 ± 3.0	< 0.0001	63.1 ± 0.4	57.6 ± 0.6	44.3 ± 1.6	< 0.0001
Fat, g/day	53.6 ± 0.6	57.4 ± 0.9	49.1 ± 3.1	0.0003	39.2 ± 0.4	41.1 ± 0.6	35.3 ± 1.5	< 0.0001
Fiber, g/day	29.5 ± 0.2	22.7 ± 0.3	15.9 ± 0.7	< 0.0001	25.6 ± 0.2	19.2 ± 0.2	13.6 ± 0.5	< 0.0001
%Energy from meal								
Breakfast	21.7 ± 0.2	7.7 ± 0.3	1.8 ± 0.7	< 0.0001	22.8 ± 0.1	11.2 ± 0.3	2.9 ± 0.6	< 0.0001
Lunch	30.6 ± 0.2	31.4 ± 0.4	24.7 ± 2.3	0.0061	30.7 ± 0.2	30.7 ± 0.4	32.2 ± 2.3	0.7826
Dinner	33.2 ± 0.2	40.8 ± 0.4	39.0 ± 2.6	< 0.0001	29.9 ± 0.2	35.0 ± 0.4	26.7 ± 2.1	< 0.0001
Snack	14.5 ± 0.2	20.1 ± 0.4	34.5 ± 1.8	< 0.0001	16.6 ± 0.2	23.1 ± 0.3	38.1 ± 1.5	< 0.0001
Eat duration, h	12.9 ± 0.03	11.4 ± 0.1	9.0 ± 0.4	< 0.0001	12.1 ± 0.02	10.4 ± 0.1	8.8 ± 0.3	< 0.0001
Meal episodes, n	5.8 ± 0.02	4.7 ± 0.04	3.5 ± 0.1	< 0.0001	5.6 ± 0.02	4.6 ± 0.02	3.8 ± 0.1	< 0.0001
%Energy from eating out	40.6 ± 0.6	46.6 ± 0.8	46.7 ± 2.8	< 0.0001	28.9 ± 0.4	35.8 ± 0.6	46.7 ± 2.3	< 0.0001
Practicing healthy diet				< 0.0001				< 0.0001
Yes	3,540 (51.4)^2)^	1,105 (35.9)	115 (42.3)		5,685 (64.1)	2,227 (52.7)	197 (52.0)	
No	2,962 (48.6)	1,802 (64.1)	151 (57.6)		2,996 (35.9)	1,752 (47.3)	167 (48.0)	

^1)^ Continuous variables are presented as means ± standard errors

^2)^ Categorical variables are presented as frequencies (weighted %)

^3)^*P*-values based on analysis of variance for continuous variables and chi-square tests for categorical variables

### Association between the frequency of main meal intake and MetS and its components

Table 3 shows the relationship between main meal frequency and the odds of MetS and its associated components. Among men, participants who consumed two main meals per day had higher odds of MetS (OR:1.16, 95% CI:1.01–1.33), abdominal obesity (OR:1.21, 95% CI:1.01–1.44), and elevated triglyceride levels (OR:1.16, 95% CI:1.04–1.29) than those who consumed three main meals per day. No significant association was detected between the frequency of main meals and MetS or its components in women.

**Table 3:** Adjusted odds ratios (ORs) and 95% confidence intervals (CIs) of MetS and its components according to main meal frequency

	Men (n = 9,675)			Women (n = 13,024)		
	Main meal frequency (meals/day)			Main meal frequency (meals/day)		
	3	2	1	3	2	1
Cases (n)/Total (n)	2,363/6,502	965/2,907	72/266	2,941/8,681	1,023/3,979	63/364
	OR (95% CI)	OR (95% CI)	OR (95% CI)	OR (95% CI)	OR (95% CI)	OR (95% CI)
MetS	1.00	1.16 (1.01–1.33)^1)^	1.07 (0.71–1.63)	1.00	1.00 (0.88–1.15)	0.77 (0.52–1.15)
Abdominal obesity	1.00	1.21 (1.01–1.44)	1.37 (0.82–2.27)	1.00	0.92 (0.78–1.08)	0.69 (0.45–1.06)
Elevated fasting blood glucose	1.00	1.00 (0.89–1.13)	1.14 (0.81–1.63)	1.00	1.01 (0.90–1.14)	0.97 (0.70–1.34)
Elevated blood pressure	1.00	1.07 (0.94–1.22)	0.99 (0.71–1.38)	1.00	1.05 (0.93–1.19)	0.84 (0.58–1.21)
Elevated triglycerides	1.00	1.16 (1.04–1.29)	1.08 (0.77–1.51)	1.00	1.13 (0.99–1.28)	1.09 (0.76–1.55)
Reduced HDL-cholesterol	1.00	0.98 (0.86–1.12)	0.98 (0.68–1.41)	1.00	1.02 (0.92–1.13)	0.91 (0.67–1.24)

OR, odds ratio; CI, confidence interval; MetS, metabolic syndrome; HDL, high-density lipoprotein

^1)^ Models were adjusted for age (years, continuous), educational level (elementary school, middle school, high school, or college), household income level (lower middle, middle, upper middle, or highest), occupation (yes or no), marriage (married or single), household type (single-person or multi-person), region (urban or rural) alcohol consumption (none, moderate, or high), smoking status (never, past, or current), regular physical activity (yes or no), total energy intake (kcal/day, continuous), and body mass index (kg/m^2^, continuous)

### Association between skipping patterns of main meals and MetS and its components

Table 4 shows the relationship between the types of main meals skipped and MetS and its associated components. Men who skipped breakfast had increased odds of MetS (OR:1.22, 95% CI:1.04–1.43), abdominal obesity (OR:1.28, 95% CI:1.05–1.56), and elevated triglycerides (OR:1.20, 95% CI:1.05–1.36) compared to those who consumed three main meals per day. Men who only consumed breakfast and dinner during the day had higher odds of elevated fasting blood glucose levels (OR:4.74, 95% CI:1.39–16.13; OR:1.54, 95% CI:1.00–2.37) than those who consumed three meals per day. Among women, compared with those who consumed three main meals per day, those who skipped breakfast had increased odds of having elevated fasting blood glucose levels (OR:1.18, 95% CI:1.02–1.35), elevated triglycerides (OR:1.19, 95% CI:1.02–1.39), and a reduced HDL-cholesterol level (OR:1.14, 95% CI:1.00–1.29) compared to those who consumed three main meals per day. However, women who skipped dinner had lower odds of elevated fasting blood glucose levels (OR:0.74, 95% CI:0.58–0.94) than those who consumed three main meals per day.

**Table 4:** Adjusted odds ratios (ORs) and 95% confidence intervals (CIs) of MetS and its associated components according to types of main meal skipped

	Main meal frequency (meals/day)						
	3	2			1		
	Breakfast, lunch, and dinner	Lunch and dinner	Breakfast and dinner	Breakfast and lunch	Breakfast	Lunch	Dinner
Men							
Cases(n)/Total(n)	2,363/6,502	660/2,128	220/568	85/211	4/11	41/114	27/141
	OR (95% CI)	OR (95% CI)	OR (95% CI)	OR (95% CI)	OR (95% CI)	OR (95% CI)	OR (95% CI)
MetS	1.00	1.22 (1.04–1.43)^1)^	0.98 (0.77–1.25)	1.09 (0.73–1.63)	4.61 (0.96–22.06)	1.33 (0.72–2.46)	0.82 (0.47–1.44)
Abdominal obesity	1.00	1.28 (1.05–1.56)	1.11 (0.81–1.53)	0.85 (0.49–1.47)	0.72 (0.13–3.90)	1.48 (0.78–2.79)	1.32 (0.60–2.89)
Elevated fasting blood glucose	1.00	1.04 (0.91–1.19)	0.91 (0.73–1.13)	0.92 (0.65–1.29)	4.74 (1.39–16.13)	0.69 (0.40–1.20)	1.54 (1.00–2.37)
Elevated blood pressure	1.00	1.05 (0.91–1.22)	1.16 (0.92–1.45)	1.04 (0.74–1.48)	1.39 (0.36–5.29)	0.84 (0.53–1.34)	1.10 (0.70–1.73)
Elevated triglyceride	1.00	1.20 (1.05–1.36)	0.99 (0.81–1.22)	1.25 (0.89–1.76)	2.37 (0.73–7.68)	1.29 (0.81–2.04)	0.90 (0.57–1.43)
Reduced HDL-cholesterol	1.00	1.01 (0.87–1.18)	0.92 (0.74–1.15)	0.81 (0.55–1.21)	0.63 (0.13–3.00)	1.02 (0.61–1.72)	0.98 (0.57–1.67)
Women							
Cases(n)/Total(n)	2,941/8,681	520/2,441	313/930	190/608	12/34	29/188	22/142
	OR (95% CI)	OR (95% CI)	OR (95% CI)	OR (95% CI)	OR (95% CI)	OR (95% CI)	OR (95% CI)
MetS	1.00	1.13 (0.95–1.35)	0.87 (0.69–1.09)	0.89 (0.68–1.16)	0.44 (0.18–1.07)	0.69 (0.40–1.20)	1.08 (0.59–1.99)
Abdominal obesity	1.00	0.99 (0.81–1.21)	0.77 (0.58–1.01)	0.91 (0.66–1.25)	0.34 (0.06–2.05)	0.63 (0.34–1.15)	0.87 (0.45–1.67)
Elevated fasting blood glucose	1.00	1.18 (1.02–1.35)	0.91 (0.74–1.12)	0.74 (0.58–0.94)	1.10 (0.37–3.22)	1.02 (0.66–1.57)	0.92 (0.55–1.52)
Elevated blood pressure	1.00	1.08 (0.92–1.26)	1.14 (0.92–1.43)	0.87 (0.67–1.12)	1.37 (0.51–3.72)	0.89 (0.53–1.49)	0.65 (0.35–1.23)
Elevated triglyceride	1.00	1.19 (1.02–1.39)	1.08 (0.86–1.34)	1.01 (0.79–1.30)	0.50 (0.20–1.26)	0.95 (0.59–1.52)	1.49 (0.90–2.47)
Reduced HDL-cholesterol	1.00	1.14 (1.00–1.29)	0.85 (0.71–1.02)	0.90 (0.72–1.12)	0.45 (0.18–1.13)	0.87 (0.59–1.29)	1.11 (0.70–1.76)

OR, odds ratio; CI, confidence interval; MetS, metabolic syndrome; HDL, high-density lipoprotein

^1)^ Models were adjusted for age (years, continuous), educational level (elementary school, middle school, high school, or college), household income level (lower middle, middle, upper middle, or highest), occupation (yes or no), marriage (married or single), household type (single-person or multi-person), region (urban or rural) alcohol consumption (none, moderate, or high), smoking status (never, past, or current), regular physical activity (yes or no), total energy intake (kcal/day, continuous), and body mass index (kg/m^2^, continuous)

## Discussion

In the present study, we found that the frequency of main meals and patterns of skipping meals were notable factors affecting the prevalence of MetS. Korean men who consumed two main meals per day had higher odds of MetS and its associated components than those who consumed three main meals per day. This association was particularly significant in those who skipped breakfast. Korean women who consumed two main meals per day and skipped breakfast had increased odds of elevated fasting blood glucose and triglyceride levels and reduced HDL cholesterol levels. However, skipping dinner was associated with reduced odds of having elevated fasting blood glucose levels among women.

Although high meal frequency is typically considered beneficial for maintaining metabolic health, previous studies assessing the association between meal frequency and metabolic health have reported inconsistent results. In a study evaluating Korean adults, men who consumed fewer than two meals per day had higher odds of MetS than those who consumed three meals per day [12]. In an Australian study by Smith et al. [40], a higher meal frequency was associated with a lower risk of MetS components such as excessive waist circumference, elevated fasting blood glucose levels, and elevated triglyceride levels among men. Additionally, eating fewer than two meals per day has been associated with higher calorie consumption per meal, resulting in a rapid increase in postprandial glucose levels, which induces an enhanced insulin response and eventually leads to insulin resistance [41]. Postprandial hyperglycemia and hyperlipidemia contribute to oxidative stress and chronic inflammation, resulting in MetS [42, 43]. However, some studies have reported that reducing the frequency of meal consumption can reduce the risk of chronic diseases. A reduction in meal frequency due to intermittent fasting was shown to be significantly associated with a decreased risk of cardiovascular disease and diabetes [44–46]. Furthermore, eating one meal per day for eight weeks was shown to reduce weight with no significant changes in serum lipid, glucose, or insulin levels compared to participants who consumed three meals per day. However, it remains unclear whether the effect of reduced meal frequency can be attributed to calorie restriction or fasting itself [47]. Despite adjusting for total energy intake, the present study did not find any notable association between meal frequency and metabolic health, possibly due to the small number of participants consuming one meal per day compared to those consuming two or three meals per day.

As the frequency of daily main meal intake decreased in men, the ratio of snack intake to healthy eating habits also decreased, increasing the odds of MetS. The results of the previous studies support this hypothesis. Previous studies have shown that the consumption of candy and chocolate increases the risk of MetS by more than 30% [48]. In addition, university students who consumed snacks had a high energy intake and a 15.36 times higher risk of having two or more MetS risk factors [49]. Studies have revealed that practicing a healthy diet lowers the risk of MetS. A previous study has shown that Koreans who adhere to healthy diets, including adequate fat intake, reduced sodium intake, and sufficient fruit and vegetable intake, have a lower risk of abdominal obesity and MetS [50].

We found no association between the frequency of consuming main meals and the odds of MetS among women. Similarly, previous studies reported no relationship between meal frequency and MetS, obesity, or body composition among women [12, 51, 52] Furthermore, studies assessing premenopausal women found no association between body fat distribution and the frequency of meal intake [53, 54]. In the present study, men with a high meal frequency had a low BMI despite high total energy intake. However, women with a high meal frequency had higher BMI and total energy intake than women with a low meal frequency. This difference between men and women might be explained by lifestyle differences, such as a higher proportion of heavy drinkers and current smokers among men than among women, along with sex-specific endocrine factors [55].

The findings of the present study demonstrate that skipping breakfast could increase the odds of MetS and its associated components in both men and women. Our findings are similar to those of a study assessing Korean adults aged 20–64 years, which reported that individuals who skipped breakfast, and had a high fat intake were more likely to have obesity and MetS [56]. Similarly, a study evaluating US adults aged 18–30 years found that participants who consumed breakfast had lower odds of weight gain, low HDL cholesterol levels, elevated blood pressure, and MetS than those who skipped breakfast [57, 58]. In a study conducted in Japan, skipping breakfast was linked to an increased risk of MetS and hypertension in men [59]. Breakfast consumption may help manage and prevent MetS, likely due to the differences in the types of nutrients and food groups consumed between breakfast eaters and those who skip breakfast. Participants who ate breakfast consumed a variety of nutrients compared to those who skipped breakfast. Those who skipped breakfast were more likely to consume less than the estimated average requirement for various nutrients such as calcium, vitamin C, and folate. Individuals who skip breakfast have a significantly higher percentage of energy from fat and a lower percentage of energy from carbohydrates [60]. Men who skip breakfast have insufficient nutrients and an imbalanced macronutrient intake [61]. Differences in the types of food consumed at dinner were observed depending on whether breakfast was consumed or skipped. A previous study has shown that individuals who skipped breakfast frequently consumed cookies, cakes, and meat at dinner [60]. An imbalanced diet, caused by skipping breakfast during the day, may increase the prevalence of MetS and its associated components.

The present study revealed that skipping dinner was associated with reduced odds of hyperglycemia among Korean women. Skipping dinner has been significantly associated with hyperglycemic blood glucose and insulin levels [29, 62]. In patients with diabetes aged 30–70 years, consuming two meals per day, that is, breakfast and lunch, was found to be a more effective strategy for managing fasting plasma glucose, C-peptide, glucagon, and insulin sensitivity levels than consuming six meals per day [62]. Furthermore, skipping dinner resulted in fewer metabolic disorders than skipping breakfast [26–29]. Even with the same quantity and quality of meals, skipping dinner results in a higher total energy expenditure per day than skipping breakfast, resulting in higher energy expenditure due to increased involuntary physical activity, even without physical activity, with a small increase in blood glucose levels and insulin response through biorhythms [29]. Differences in nutrient intake patterns based on meal-skipping patterns have also been documented. Kahleova et al. [62] showed that saturated fatty acid intake significantly decreased on days when individuals consumed only breakfast and lunch and skipped dinner. A reduced intake of saturated fatty acids improves insulin action and is associated with a low risk of insulin resistance and type 2 diabetes [62].

This study has several strengths. Firstly, to the best of our knowledge, this is the first study to investigate the association between the frequency of daily main meal intake and meal-skipping patterns with the odds of MetS and its associated components among Korean adults. Secondly, statistical analytical models were adjusted for potential confounding variables such as socioeconomic and health-related behavioral variables, and total energy intake, allowing for the identification of an independent association between main meal skipping patterns and MetS. However, this study also has limitations. Firstly, given the nature of the cross-sectional study design, the main meal frequency, skipping patterns, and MetS risk variables were evaluated simultaneously, and the mechanisms underlying their association could not be explained since there was no evidence of a temporal relationship between the independent and dependent variables. Second, there may be a potential recall bias because dietary information was collected using self-report questionnaires. Finally, despite adjusting for socio-demographic variables, the influence of unmeasured or unreviewed variables cannot be excluded. For example, we did not adjust for sex-specific biological factors or the composition of dietary intake, which may have influenced our results.

## Conclusions

In conclusion, among Korean men, the consumption of two main meals per day increased the odds of MetS and its associated components compared to the consumption of three main meals per day, whereas skipping breakfast enhanced the odds of MetS in both men and women. These findings suggest that the number of main meals consumed can affect MetS, but skipping main meals has a greater impact on the prevalence of MetS. Given that skipping breakfast can be positively associated with MetS and its associated components, nutritional education should be provided to emphasize the importance of breakfast consumption in optimizing metabolic health. Prospective cohort and intervention studies are required to explore the causal relationships between the frequency and skipping patterns of main meal consumption.

## Data Availability

The datasets generated and analyzed in the current study are available from the Korea National Health and Nutrition Examination Survey (KNHANES) repository, https://knhanes.kdca.go.kr/knhanes/sub03/sub03_02_05.do (accessed on December 8, 2022).

## Abbreviations

ANOVA: Analysis of variance
ATP: Adult Treatment Panel
BMI: Body mass index
CI: Confidence interval
HDL: High-density lipoprotein
KNHANES: Korea National Health and Nutrition Examination Survey
MetS: Metabolic syndrome
NCEP: National Cholesterol Education Program
NFBC: Northern Finland Birth Cohort
OR: Odds ratio
WHO: World Health Organization

## References

[1] Kim MK, Park JH (2012). Metabolic syndrome. J Korean Med Assoc.

[2] Alberti KGMM, Zimmet PZ (1998). Definition, diagnosis and classification of diabetes mellitus and its complications. Part 1: diagnosis and classification of diabetes mellitus. Provisional report of a WHO consultation. Diabet Med.

[3] Grundy SM (2002). Third report of the national cholesterol Education Program (NCEP) Expert Panel on detection, evaluation, and treatment of high blood cholesterol in adults (Adult Treatment Panel III) final report. Circulation.

[4] Huang PL (2009). A comprehensive definition for metabolic syndrome. Dis Models Mech.

[5] Zimmet P, Magliano D, Matsuzawa Y, Alberti G, Shaw J (2005). The metabolic syndrome: a global public health problem and a new definition. J Atheroscler Thromb.

[6] Yoon K-H, Lee J-H, Kim J-W, Cho JH, Choi Y-H, Ko S-H, Zimmet P, Son H-Y (2006). Epidemic obesity and type 2 diabetes in Asia. Lancet.

[7] Huh JH, Lee JH, Moon JS, Sung KC, Kim JY, Kang DR. Metabolic syndrome severity score in Korean adults: analysis of the 2010–2015 Korea National Health and Nutrition Examination Survey. J Korean Med Sci. 2019, 34. 10.3346/jkms.2019.34.e48 PMC6374550 30787681

[8] Jang YS, Joo HJ, Jung YH, Park E-C, Jang S-Y (2022). Association of the “Weekend Warrior” and other physical activity patterns with metabolic syndrome in the south korean Population. Int J Environ Res Public Health.

[9] Grundy SM, Hansen B, SmithJr SC, Cleeman JI, Kahn RA. Participants fC. Clinical management of metabolic syndrome: report of the American Heart Association/National Heart, Lung, and Blood Institute/American Diabetes Association conference on scientific issues related to management. Arterioscler Thromb Vasc Biol. 2004, 24:e19-e24. 10.1161/01.ATV.0000112379.88385.67 14766740

[10] Nguyen HT (2022). Effect of nutrition intervention on the prevalence of metabolic syndrome at Kon Tum General Hospital, Vietnam. J Complement Med Res.

[11] Storz MA (2020). The role of vegan diets in lipotoxicity-induced beta-cell dysfunction in type-2-diabetes. J Popul Ther Clin Pharmacol.

[12] Jung C-H, Lee JS, Ahn HJ, Choi J-S, Noh MY, Lee JJ, Lee EY, Lim JH, Lee YR, Yoon SY (2017). Association of meal frequency with metabolic syndrome in korean adults: from the Korea National Health and Nutrition Examination Survey (KNHANES). Diabetol Metab Syndr.

[13] Carlson O, Martin B, Stote KS, Golden E, Maudsley S, Najjar SS, Ferrucci L, Ingram DK, Longo DL, Rumpler WV (2007). Impact of reduced meal frequency without caloric restriction on glucose regulation in healthy, normal-weight middle-aged men and women. Metab.

[14] Titan SMO, Bingham S, Welch A, Luben R, Oakes S, Day N, Khaw K-T (2001). Frequency of eating and concentrations of serum cholesterol in the Norfolk population of the european prospective investigation into cancer (EPIC-Norfolk): cross sectional study. Br Med J.

[15] Jääskeläinen A, Schwab U, Kolehmainen M, Pirkola J, Järvelin M-R, Laitinen J (2013). Associations of meal frequency and breakfast with obesity and metabolic syndrome traits in adolescents of Northern Finland Birth Cohort 1986. Nutr Metab Cardiovasc Dis.

[16] Sierra-Johnson J, Undén A-L, Linestrand M, Rosell M, Sjogren P, Kolak M, Faire UD, Fisher RM (2008). Hellénius M-L. Eating meals irregularly: a novel environmental risk factor for the metabolic syndrome. Obes.

[17] Pliquett RU, Führer D, Falk S, Zysset S, Cramon DYv, Stumvoll M (2006). The effects of insulin on the central nervous system-focus on appetite regulation. Horm Metab Res.

[18] Kim KH (2010). Food habits, eating behaviors and food frequency by gender and among Seoul and other regions in upper-grade elementary school children. KJCN.

[19] Kim M-J, Kim Y-H (2010). Dietary habits, nutrition knowledge and dietary behaviors of the 3rd grade elementary school students in Ulsan area by sex and skipping breakfast. J East Asian Soc Diet Life.

[20] Yi B-S, Yang I-S (2006). An exploratory study for identifying factors related to breakfast in elementary, middle and high school students. Korean J Community Nutr.

[21] Jung J, Kim A-S, Ko H-J, Choi H-I, Hong H-E (2020). Association between breakfast skipping and the metabolic syndrome: the Korea National Health and nutrition examination survey, 2017. Med.

[22] Koletzko B, Toschke AM (2010). Meal patterns and frequencies: do they affect body weight in children and adolescents?. Crit Rev Food Sci Nutr.

[23] Veltsista A, Laitinen J, Sovio U, Roma E, Järvelin M-R, Bakoula C (2010). Relationship between eating behavior, breakfast consumption, and obesity among finnish and greek adolescents. J Nutr Educ Behav.

[24] Smith KJ, Gall SL, McNaughton SA, Blizzard L, Dwyer T, Venn AJ (2010). Skipping breakfast: longitudinal associations with cardiometabolic risk factors in the Childhood Determinants of Adult Health Study. Am J Clin Nutr.

[25] Timlin MT, Pereira MA (2007). Breakfast frequency and quality in the etiology of adult obesity and chronic diseases. Nutr Rev.

[26] Allison KC, Hopkins CM, Ruggieri M, Spaeth AM, Ahima RS, Zhang Z, Taylor DM, Goel N (2021). Prolonged, controlled daytime versus delayed eating impacts weight and metabolism. Curr Biol.

[27] Bo S, Fadda M, Castiglione A, Ciccone G, Francesco AD, Fedele D, Guggino A, Caprino MP, Ferrara S, Boggio MV (2015). Is the timing of caloric intake associated with variation in diet-induced thermogenesis and in the metabolic pattern? A randomized cross-over study. Int J Obes Suppl.

[28] Jakubowicz D, Barnea M, Wainstein J, Froy O (2013). High caloric intake at breakfast vs. dinner differentially influences weight loss of overweight and obese women. Obes.

[29] Nas A, Mirza N, Hägele F, Kahlhöfer J, Keller J, Rising R, Kufer TA, Bosy-Westphal A (2017). Impact of breakfast skipping compared with dinner skipping on regulation of energy balance and metabolic risk. Am J Clin Nutr.

[30] Nuvoli G (2015). Family meal frequency, weight status and healthy management in children, young adults and seniors. A study in Sardinia. Italy Appetite.

[31] Aemro M, Mesele M, Birhanu Z, Atenafu A. Dietary diversity and meal frequency practices among infant and young children aged 6–23 months in Ethiopia: a secondary analysis of Ethiopian demographic and health survey 2011. J Nutr Metab. 2013, 2013. 10.1155/2013/782931 PMC3878383 24455218

[32] Silva FA, Candiá SM, Pequeno MS, Sartorelli DS, Mendes LL, Oliveira R, Netto MP, Cândido APC (2017). Daily meal frequency and associated variables in children and adolescents. J Pediatr.

[33] Lee S-K (2008). Acculturation, meal frequency, eating-out, and body weight in Korean Americans. Nutr Res Pract.

[34] Egli V, Hunter L, Roy R, Te Morenga L, De Backer C, Teunissen L, Cuykx I, Decorte P, Gerritsen S. Household Mealtimes During the 2020 COVID-19 Lockdown in Aotearoa New Zealand: The Influence of Household Type and Psychological Distress. Front nutr. 2022, 9. 10.3389/fnut.2022.855866 PMC9237537 35774541

[35] White J, Halliwell E (2011). Family meal frequency and alcohol and tobacco use in adolescence: testing reciprocal effects. J Early Adolesc.

[36] Mota J, Fidalgo F, Silva R, Ribeiro JC, Santos R, Carvalho J, Santos MP (2008). Relationships between physical activity, obesity and meal frequency in adolescents. Ann Hum Biol.

[37] Westerterp-Plantenga M, Kovacs E, Melanson K (2002). Habitual meal frequency and energy intake regulation in partially temporally isolated men. Int J Obes Suppl.

[38] Bellisle F, McDevitt R, Prentice AM (1997). Meal frequency and energy balance. Br J Nutr.

[39] User Guide for the Fourth Korea National Health. and Nutrition Examination Survey (KNHANES VII); Korea Centers for Disease Control and Prevention: Cheongwon, Korea. Accessed.

[40] Smith KJ, Blizzard L, McNaughton SA, Gall SL, Dwyer T, Venn AJ (2012). Daily eating frequency and cardiometabolic risk factors in young australian adults: cross-sectional analyses. Br J Nutr.

[41] Basciano H, Federico L, Adeli K (2005). Fructose, insulin resistance, and metabolic dyslipidemia. Nutr Metab.

[42] Ceriello A, Bortolotti N, Motz E, Pieri C, Marra M, Tonutti L, Lizzio S, Feletto F, Catone B, Taboga C (1999). Meal-induced oxidative stress and low-density lipoprotein oxidation in diabetes: the possible role of hyperglycemia. Metab.

[43] Ceriello A, Motz E (2004). Is oxidative stress the pathogenic mechanism underlying insulin resistance, diabetes, and cardiovascular disease? The common soil hypothesis revisited. Arterioscler Thromb Vasc Biol.

[44] Longo VD, Mattson MP (2014). Fasting: molecular mechanisms and clinical applications. Cell Metab.

[45] RuiqianWan PMattsonM (2005). Beneficial effects of intermittent fasting and caloric restriction on the cardiovascular and cerebrovascular systems. J Nutr Biochem.

[46] Varady KA, Hellerstein MK (2007). Alternate-day fasting and chronic disease prevention: a review of human and animal trials. Am J Clin Nutr.

[47] Anson RM, Guo Z, Cabo Rd, Iyun T, Rios M, Hagepanos A, Ingram DK, Lane MA, Mattson MP (2003). Intermittent fasting dissociates beneficial effects of dietary restriction on glucose metabolism and neuronal resistance to injury from calorie intake. Proc Natl Acad Sci U S A.

[48] Mirmiran P, Aghayan M, Bakhshi B, Hosseinpour-Niazi S, Azizi F (2021). Socioeconomic status and lifestyle factors modifies the association between snack foods intake and incidence of metabolic syndrome. Nutr J.

[49] Vergetaki A, Linardakis M, Papadaki A, Kafatos A (2011). Presence of metabolic syndrome and cardiovascular risk factors in adolescents and University students in Crete (Greece), according to different levels of snack consumption. Appetite.

[50] Bae Y-J (2016). Relationship among practicing healthy diet and metabolic syndrome indicators in adults-from the Korea National Health and Nutrition Examination Survey, 2013 ~ 2014. J Nutr Health.

[51] Yannakoulia M, Melistas L, Solomou E, Yiannakouris N (2007). Association of eating frequency with body fatness in pre-and postmenopausal women. Obes.

[52] Drummond S, Crombie N, Cursiter M, Kirk T (1998). Evidence that eating frequency is inversely related to body weight status in male, but not female, non-obese adults reporting valid dietary intakes. Int J Obes.

[53] Duval K, Strychar I, Cyr M-J, Prud’homme D, Rabasa-Lhoret R, Doucet É (2008). Physical activity is a confounding factor of the relation between eating frequency and body composition. Am J Clin Nutr.

[54] CD S, RC M (1996). Relationship between feeding pattern and body mass index in 220 free-living people in four age groups. Eur J Clin Nutr.

[55] Kautzky-Willer A, Harreiter J, Pacini G (2016). Sex and gender differences in risk, pathophysiology and complications of type 2 diabetes mellitus. Endocr Rev.

[56] Chung S-J, Lee Y, Lee S, Choi K (2015). Breakfast skipping and breakfast type are associated with daily nutrient intakes and metabolic syndrome in korean adults. Nutr Res Pract.

[57] Deshmukh-Taskar R, A.Nicklas P, O’Neil T, Keast CE, Radcliffe DR, Cho JD (2010). The relationship of breakfast skipping and type of breakfast consumption with nutrient intake and weight status in children and adolescents: the National Health and Nutrition Examination Survey 1999–2006. J Am Diet Assoc.

[58] Odegaard AO, David R, Jacobs J, Steffen LM, Horn LV, Ludwig DS, Pereira MA (2013). Breakfast frequency and development of metabolic risk. Diabetes Care.

[59] Katsuura-Kamano S, Arisawa K, Uemura H, Nguyen TV, Takezaki T, Ibusuki R, Suzuki S, Otani T, Okada R, Kubo Y (2021). Association of skipping breakfast and short sleep duration with the prevalence of metabolic syndrome in the general japanese population: baseline data from the Japan Multi-Institutional collaborative cohort study. Prev Med Rep.

[60] Min C, Noh H, Kang Y-S, Sim HJ, Baik HW, Song WO, Yoon J, Park Y-H, Joung H (2011). Skipping breakfast is associated with diet quality and metabolic syndrome risk factors of adults. Nutr Res Pract.

[61] Lee S, Shim J, Kim J, Moon H (1996). The effect of breakfast regularity on eating habits, nutritional and health status in adults. Korean J Nutr.

[62] Kahleova H, Belinova L, Malinska H, Oliyarnyk O, Trnovska J, Skop V, Kazdova L, Dezortova M, Hajek M, Tura A (2014). Eating two larger meals a day (breakfast and lunch) is more effective than six smaller meals in a reduced-energy regimen for patients with type 2 diabetes: a randomised crossover study. Diabetologia.

